# Supplementary material for: A value creation model from science-society interconnections: Archetypal analysis combining publications, survey and altmetric data
Source: PLoS One. 2022 Jun 3;17(6):e0269004. doi: 10.1371/journal.pone.0269004 (PMC9165788; doi:10.1371/journal.pone.0269004)
Supplement: Attachment — Submitted filename: Response to reviews_FINAL.pdf [file pone.0269004.s001.pdf]

Dear dr. Lutz Bornmann,

Thank you for the opportunity to respond to the two reviews of our manuscript PONE-D-21-16293: ‘A value creation model from science-society interconnections: Components and archetypes’. We are grateful to both reviewers for their constructive comments and suggestions. We also appreciate the reviewers’ willingness to engage fully with the paper, which is a slightly unorthodox mix of conceptual development, methodological experiment, and empirical trial, and for their overall support for our work.

In the following we address, first, the major general concern that both reviewers had with the manuscript. Second, we respond to the specific requests for clarification or change from each reviewer.

The major concern of both reviews is the quality of those data used as proxies for the components in our conceptual model. The reviewers also note that we are not naïve to this issue in the presentation of our work. As they also acknowledge, we make clear cautionary statements about both the quality of our proxies and our desire to improve them in the future, and regarding the potentially considerable margin for improvement in the approximations of our archetypes that better data may produce. The reviewers suggest remedies for this concern related to how the paper is framed. Reviewer #1 would like the paper to be more prominently framed as exploratory in its objectives. Reviewer #2 would like the title changed to reflect archetypes as being our method not our output, more discussion of the limitations of the most used data sources, and if possible, suggestions about new relevant data sources.

In response to these requests, we have added two statements regarding our work as exploratory research, one in the introduction and one in the discussion section. We have changed the title, to refer to archetypes as our method, not as a consolidated empirical output. The discussion of the limitations of the paper, particularly the data proxies, has been strengthened, although we do not wish to speculate about potential future data sources, as to the best of our knowledge such alternatives are currently scarce, with the obvious major exception of the Overton database we included in the manuscript.

In summary, we agree with the main concern of the reviewers, but would also contend that this issue would have been far more serious if the empirical results we obtained did not appear intuitively reasonable and did not allow for coherent interpretation according to our model. Neither reviewer raised any concerns about the ‘configurations’ that emerged from our use of the archetype method. Indeed, we would not have submitted this exploratory research if we did not believe that our results were coherent with our expectations and our conceptual model. Accordingly, we consider our empirical results a proof of concept or exploratory step in the right direction, which will inevitably be improved upon through access to better data proxies and other potential design modifications that we also describe in the final section of the manuscript – such as the potential amenability of the model and method to ‘big data’ approaches. In any case, we would like to point out that putting together a dataset with information from different sources (self-reported, publication and secondary databases) is rare and very few studies can be found in this regard.

The specific comments, requests for clarification of changes from Reviewer #1 included:

- *In lines 234-236, the authors state: "As represented in Figure 1, we understand research value to be generated actively through the multiple research-based knowledge processes that produce and entwine science and society." I do not see that Figure 1 is a good representation for this statement. I do not find Figure 1 helpful in its current form.*

Figure 1 has been deleted and other figures renumbered.

- *Regarding the data (lines 330-343) I wonder if "records" (n=6,174) in line 342 refers to "publications" or some other kind of records. If "records" refers to "publications", a very low percentage of the total amount of publications retrieved (n=83,551) could be analyzed. Even if "records" refers to "respondents" (n=11,419), just more than half of the respondents data were analyzed*

We now refer to n=6,174 records as survey records, as they are instances of survey responses and their aggregated publication, OA and altmetric indicators.

- *In line 363, the authors state that they used R for analysis. Usage of R should be cited. For increasing reproducibility and benefit for the readers, I think that the authors should share their R scripts as supplemental information*

The R scripts have been supplied and R has been cited.

- *The description of the identification of archetypes is not clear enough*

We have expanded the section on statistical design. Specifically, we have described how archetypes are assigned to observations and how these are interpreted.

- *Figures 4-7 contain VOSviewer overlay maps on the right panels. The color codes are explained only in part. It is explained what blue and yellow colors mean but not the colors in-between. However, I think that the explanation of the colors is inaccurate.*
- *It puzzles me that the scales have different ranges of values. I suppose that the value of zero corresponds to the absence of a term. What does a value of one mean? Are these terms present in all publication titles? Does a value of 0.5 mean that the term is present in 50% of the publication titles?*

We have now included a brief explanation of the grading and maximum and minimum values in order to ease the interpretation of the overlay maps.

- *It would be helpful for a detailed inspection of Figures 4-7 if web-startable links or the map files of the VOSviewer overlay maps were provided as supplemental information.*

Thank you for the suggestion. These online versions are now available and have been mentioned in each figure’s caption.

- *Reference 51 contains an invalid DOI*

Many thanks. This has been corrected and the rest of the references have also been revised accordingly.

The specific comments, requests for clarification of changes from Reviewer #2 included:

- *The introduction of C assumes that summing up numbers of categorized individual activities of Spanish researchers can provide information about how science-society relations are organized in Spain. Can they tell us how clinical research interacts with clinical practice in the health services? Can they tell us how educational research and its organizations in Spain interact with the Spanish school system and its organizations? Can they tell us how geophysics, in an organized way, comes to the aid in the crisis of a volcano outbreak? I think the survey results can only tell us about numbers of pre-categorized activities. Can configurations of field-related typical interactions emerge from this?*

Our data are presented at the broad field level and show the configurations of relations between the different components in our model. These data may be useful for some of the other research questions listed here, although they are not intended for generalization of the Spanish system. Survey results do record numbers of 'pre-categorized activities' (i.e., our variable dimensions). There is a rich literature examining configuring configurations of these interactions (which we draw on in part of our model development) that does present results of typical common channels of interactions. The model and analysis presented in our manuscript is complementary to some of these types of studies but is also very different from them.

- *The study forgets that most publications representing the societal interactions of the same researchers will not be in WoS and they will be in the Spanish language if in publicly available writing at all. The use of WoS-publications and mentions of them in social media is a very limited representation of societal interaction. ... The trouble is that the study is bound up with documents with DOI – we are inside the English-speaking international academic publishing and library world*

It is true that the vast majority of the publications we used are written in English. They are also the most important article publications of many of these researchers as, for reasons beyond our control, English remains the hegemonic publication language in most globalized fields of science (certainly in STEM and BIOMED). We agree with the reviewer that there are limitations in terms of language and coverage associated with using the WoS (http://doi.org/10.1023/A:1010549719484) and that improvement in publication databases would enhance the quality of research outputs using these sources. The data proxies for our components, however, cannot inform about representativeness in a given context but they reflect value creation processes through different forms of science-society interconnections.

- *[P]olicy impact is almost invisible in the results*

The data proxy used for the public policy value component is limited, as we state clearly in the manuscript. We have also strengthened our acknowledgement of the limitations of the data proxies used overall. The emergence of the new Overton database on policy documents, which we also mentioned, is a promising opportunity that we intend to exploit in the future to improve the recognition of policy take-up in our analyses.

We appreciate reviewers' comments and suggestions, which we have incorporated in the new version of the manuscript.

Sincerely,

The authors
