# Supplementary material for: A value creation model from science-society interconnections: Archetypal analysis combining publications, survey and altmetric data
Source: PLoS One. 2022 Jun 3;17(6):e0269004. doi: 10.1371/journal.pone.0269004 (PMC9165788; doi:10.1371/journal.pone.0269004)
Supplement: Attachment — Submitted filename: rebuttal_letter.pdf [file pone.0269004.s002.pdf]

REBUTTAL LETTER

Manuscript ID: PONE-D-21-16293R1
Title: A value creation model from science-society interconnections: Archetypal analysis combining publications, survey and altmetric data
Authors: Irene Ramos-Vielba, Nicolas Robinson-Garcia and Richard Woolley

March 18, 2022
Lutz Bornmann
Academic Editor, Plos One

Dear Dr. Bornmann,

Thank you for supervising the review process of this manuscript. We would like to acknowledge especially the thorough work of the reviewers who have pushed us to revise our working processes and ensure the reproducibility and transparency of the present study.

We would like to apologize for the delay of this new version, but the comments made by reviewer #1 lead us to not only correct the issues they pointed out, but to revise the entire analysis as well as update the dataset. This has allowed us to enlarge our sample to over 9,000 cases. The updated dataset is available at https://zenodo.org/record/6393226. Furthermore, a codebook has been published following the whole production of the Archetypal Analysis (https://rpubs.com/elrobin/value-components), by publishing it in RPubs we also ensure that no bugs can be encountered, as the code is rendered in order to produce the codebook.

The major changes we observe with regard to the previous version is that the archetypes seem to be more robust across fields, in the case of Social Sciences and Humanities now identifying three archetypes instead of two. While the model and most of the conclusions reached remain the same, the methods and results sections have been modified accordingly in order to accommodate the changes observed in the new figures produced. Finally, the online figures have also been updated accordingly.

Once again, we would like to thank both the editor as well as the two anonymous reviewers for such an enriching process which has undoubtedly improved the manuscript.

Sincerely,

The Authors
